# Novel Nested-Seq Approach for SARS-CoV-2 Real-Time Epidemiology and In-Depth Mutational Profiling in Wastewater

**DOI:** 10.3390/ijms22168498

**Published:** 2021-08-07

**Authors:** Margaritis Avgeris, Panagiotis G. Adamopoulos, Aikaterini Galani, Marieta Xagorari, Dimitrios Gourgiotis, Ioannis P. Trougakos, Nikolaos Voulgaris, Meletios-Athanasios Dimopoulos, Nikolaos S. Thomaidis, Andreas Scorilas

**Affiliations:** 1 Department of Biochemistry and Molecular Biology, Faculty of Biology, National and Kapodistrian University of Athens, 15771 Athens, Greece; margaritis.avgeris@gmail.com (M.A.); padamopoulos@biol.uoa.gr (P.G.A.); 2 Laboratory of Clinical Biochemistry and Molecular Diagnostics, Second Department of Pediatrics, School of Medicine, National and Kapodistrian University of Athens, “P. & A. Kyriakou” Children’s Hospital, 11527 Athens, Greece; mxagor@med.uoa.gr (M.X.); dgourg@med.uoa.gr (D.G.); 3 Laboratory of Analytical Chemistry, Department of Chemistry, National and Kapodistrian University of Athens, 15771 Athens, Greece; kategalani@hotmail.gr (A.G.); ntho@chem.uoa.gr (N.S.T.); 4 Department of Cell Biology and Biophysics, Faculty of Biology, National and Kapodistrian University of Athens, 15771 Athens, Greece; itrougakos@biol.uoa.gr; 5 Division of Geophysics & Geothermics, Department of Geology & Geoenvironment, National and Kapodistrian University of Athens, 15771 Athens, Greece; voulgaris@geol.uoa.gr; 6 Department of Clinical Therapeutics, School of Medicine, National and Kapodistrian University of Athens, 11528 Athens, Greece; mdimop@med.uoa.gr

**Keywords:** COVID-19, 2019-nCov, SARS-CoV-2 mutations, SARS-CoV-2 sequencing, sewage, nested PCR, SARS-CoV-2 NGS, wastewater-based epidemiology, D614G, spike protein

## Abstract

Considering the lack of effective treatments against COVID-19, wastewater-based epidemiology (WBE) is emerging as a cost-effective approach for real-time population-wide SARS-CoV-2 monitoring. Here, we report novel molecular assays for sensitive detection and mutational/variant analysis of SARS-CoV-2 in wastewater. Highly stable regions of SARS-CoV-2 RNA were identified by RNA stability analysis and targeted for the development of novel nested PCR assays. Targeted DNA sequencing (DNA-seq) was applied for the analysis and quantification of SARS-CoV-2 mutations/variants, following hexamers-based reverse transcription and nested PCR-based amplification of targeted regions. Three-dimensional (3D) structure models were generated to examine the predicted structural modification caused by genomic variants. WBE of SARS-CoV-2 revealed to be assay dependent, and significantly improved sensitivity achieved by assay combination (94%) vs. single-assay screening (30%–60%). Targeted DNA-seq allowed the quantification of SARS-CoV-2 mutations/variants in wastewater, which agreed with COVID-19 patients’ sequencing data. A mutational analysis indicated the prevalence of D614G (S) and P323L (RdRP) variants, as well as of the Β.1.1.7/alpha variant of concern, in agreement with the frequency of Β.1.1.7/alpha variant in clinical samples of the same period of the third pandemic wave at the national level. Our assays provide an innovative cost-effective platform for real-time monitoring and early-identification of SARS-CoV-2 variants at community/population levels.

## 1. Introduction

A cluster of severe pneumonia cases of unknown origin, linked to the Huanan seafood wholesale market in Wuhan, Hubei Providence, China, were reported by the Chinese Health Authorities on 31 December 2019. Sequencing-based analysis of lower respiratory tract samples (bronchoalveolar lavage fluid) identified a novel beta-coronavirus sharing >85% sequence similarity with a bat severe acute respiratory syndrome (SARS)-like coronavirus (CoV), provisionally indicated as 2019-nCoV, to be the causative pathogen of the coronavirus disease 2019 (COVID-19) [1,2].

CoVs constitute a family of enveloped positive-strand RNA viruses of 26–32 kilobases (kb) [3]. This seventh member of the CoV family that causes disease in humans, was further characterized showing sequence homology of approximately 79% and 50% with the SARS-CoV (now referred to as SARS-CoV-1) of the 2002 outbreak in China and the Middle East respiratory syndrome (MERS)-CoV of the 2012 outbreak in the Middle East (mainly in Saudi Arabia), respectively [4]. Thereafter, this novel CoV was named SARS-CoV-2 [5], and was rapidly spread to most countries worldwide, leading to the announcement of the COVID-19 pandemic by the World Health Organization (WHO) on 12 March 2020.

Due to the lack of established herd immunity and effective therapeutic treatments [6], the identification of COVID-19 patients and asymptomatic carriers that can transmit the virus, is still being adopted worldwide as the main approach for disease monitoring and management. At the same time, the availability of SARS-CoV-2 genomic sequences have significantly improved our understanding of SARS-CoV-2 evolution and spread, supported the identification of strains with selective advantage to become the dominant strain over other variants, and provided a risk-stratification tool for SARS-CoV-2 infectivity ranging from individual patients to community/national scales [7,8]. Nonetheless, and although risk-based community/population-wide PCR screening strategies have clearly contributed to the control of epidemics, lessening of death rates, and reopening of economic activities (see Wuhan and Singapore examples [9,10]), with the current methods/resources, only symptomatic and suspicious cases are being tested and only a fraction of them is being sequenced so far in most countries, highlighting the need for population-wide screening approaches for SARS-CoV-2 spread and mutational/variant monitoring.

Wastewater-based epidemiology (WBE) has been successfully applied in numerous case studies worldwide, with the most extensive so far being the estimation of drug consumption [11,12]. Although WBE cannot replace clinical screening and diagnostics, it still offers, due to the significant lower scale of samples needed to be tested, a cheaper and faster way for population-wide surveillance without selection bias. In this regard, WBE screening could capture SARS-CoV-2 asymptomatic carriers who are less likely to undergo testing and symptomatic patients avoiding testing due to stigmatization and social isolation. Moreover, mutational profiling of SARS-CoV-2 in wastewater could represent an innovative, cost-effective approach towards the establishment of an early-warning system for the global monitoring of SARS-CoV-2 genomic epidemiology at community/population levels [13,14,15].

Nonetheless, the development and optimization of the analytical protocols needed for SARS-CoV-2 WBE are very challenging due to the nature of the sample (PCR inhibition, complexity of DNA/RNA templates), the moderate efficiency of SARS-CoV-2 concentration, and the quality/degradation of the RNA template, for example, due to excessive amounts of detergents in wastewater samples [16]. To overcome these bottlenecks, we have performed an extensive in silico RNA stability analysis to identify the most stable genomic regions of SARS-CoV-2 and we have developed four different nested PCR/real-time PCR assays in order to improve the sensitivity and reduce the false negative rate of SASR-CoV-2 detection in wastewater. Moreover, targeted DNA-seq was applied for the mutational/strain analysis of SARS-CoV-2 in wastewater, following nested PCR-based amplification of targeted regions of the SASR-CoV-2 genome.

## 2. Results

### 2.1. In Silico Analysis of SARS-CoV-2 RNA Stability

**Figure 1:**
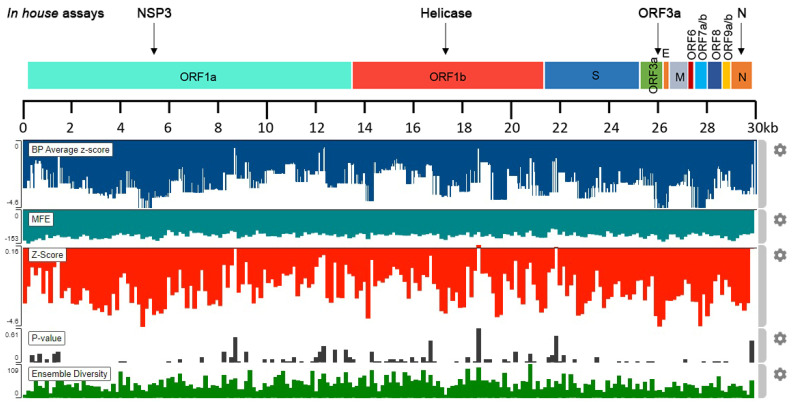
Schematic illustration of the in silico SARS-CoV-2 RNA stability analysis with the ScanFold algorithm, indicating the highly predicted regions targeted by our nested PCR/real-time PCR detection assays (NSP3, helicase, ORF3a, and N detection assays).

Bioinformatic analysis with the ScanFold server provided an informative overview regarding the highly predicted stable regions of SARS-CoV-2 RNA. Specifically, the analyses led to the identification of 300 nt sequences bearing negative z-scores, which were predicted to possess low MFE native values, thus, being more stable. Sequences with notable negative z-scores were found to distribute across the SARS-CoV-2 genome (Figure 1).

### 2.2. Nested PCR Approach Significantly Improves SARS-CoV-2 Detection Sensitivity

On the basis of the results from the in silico stability analysis, we designed four different nested PCR/real-time PCR assays on: (a) N gene, nucleocapsid phosphoprotein (N assay); (b) ORF1ab gene, helicase (helicase assay); (c) ORF1ab gene, nsp3 (NSP3 assay); (d) ORF3a gene, ORF3a protein (ORF3a assay).

To investigate the impact of our novel assays on SARS-CoV-2 detection sensitivity, serial dilutions of SARS-CoV-2 complete genome RNA control, covering nine orders of magnitude (from 10^3^ to 2.5 RNA copies/reverse transcription) were analyzed by the novel nested PCR/real-time PCR assays, and a newly in-house developed assay using the CDC proposed “2019-nCoV_N1” set of primers and probe (CDC/2019-nCoV_N1-based assay) (https://www.cdc.gov/coronavirus/2019-ncov/lab/rt-pcr-panel-primer-probes.html accessed on 1 July 2020).

Using the CDC/2019-nCoV_N1-based assay, detection of SARS-CoV-2 RNA was achieved in up to five cDNA copies/PCR reaction (weak PCR product). The application of the nested PCR/real-time PCR assays improved the detection performance in up to two cDNA copies/PCR reaction for N, helicase, and ORF3a assays. Furthermore, the NSP3 assay detected SARS-CoV-2 RNA in up to five cDNA copies/PCR reaction (Figure S1). Similar results regarding the performance and the limit of detection (LOD) on the SARS-CoV-2 RNA control sample were highlighted by the nested real-time PCR assays (Figure S2).

### 2.3. Effective SARS-CoV-2 Detection in Wastewater Requires Amplification of Multiple Targets

Our novel nested PCR/real-time PCR assays along with the CDC/2019-nCoV_N1-based assay were applied to 30 wastewater samples (S1–S30) obtained from Wastewater Treatment Plant (WWTP) of Athens from August (n = 2), September (n = 16), and October (n = 12) 2020. The cycle threshold (C_T_) values of the positive samples per assay are presented in Table 1.

**Figure 2:**
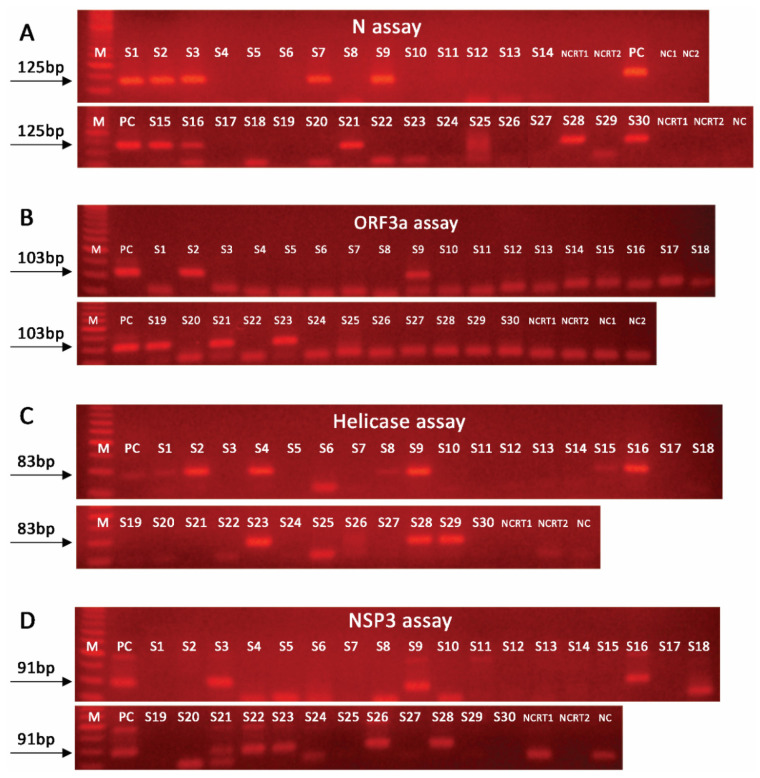
Agarose-gel electrophoresis of the PCR products of our nested PCR assays: (**A**) N assay; (**B**) ORF3a assay; (**C**) helicase assay; (**D**) NSP3 assay, in 30 wastewater samples (S1–S30). M, 50 bp DNA ladder; PC, positive control; NC, PCR negative control; NCRT, reverse transcription negative control.

SARS-CoV-2 RNA was detected, by at least one assay, in 17 samples (17/30, 56.7%), while 13 samples were negative (13/30, 43.3%). Among the 17 positive samples, the CDC/2019-nCoV_N1-based assay was positive in five samples (sensitivity 29.4%). In this study, the developed novel nested real-time PCR assays resulted in the following detection of SARS-CoV-2 RNA: (a) N assay, 10 samples (sensitivity 58.8%); (b) NSP3 assay, 9 samples (sensitivity 52.9%); (c) helicase assay, 7 samples (sensitivity 41.2%); and (d) ORF3a assay, 5 samples (sensitivity 29.4%). As expected, the application of the novel nested real-time PCR assays resulted in significantly lower CT values vs. the CDC/2019-nCoV_N1-based assay (Table 1), thus, highlighting their improved analytical performance. The analysis of the samples with the nested PCR assays (end-point analysis) led to the same results for most of the samples (Figure 2). Specifically, the agreement of nested PCR/real-time PCR results was 100% for N and ORF3a assays. A notable discrepancy was observed for one sample (S20), which was found negative by the NSP3 nested PCR assay, as well as for three samples (S1, S8, and S15) which showed a weak positive signal in the helicase nested PCR assay as compared with the same nested real-time PCR assay. In this regard, the overall agreement of nested PCR/real-time PCR assays was 96.7%.

Most importantly, in the majority of the positive samples (10/17, 58.8%), SARS-CoV-2 RNA was detected by a single assay, while five (5/17, 29.4%) and one (1/17, 5.9%) samples were scored positive by three or four assays, respectively. In this regard, a significantly improved detection specificity was achieved by the following combinations: (a) N and NSP3 assays, 14/17, sensitivity 82.4%; (b) N, NSP3, and helicase assays, 16/17, sensitivity 94.1%. Our results clearly highlight the significantly improved sensitivity by the combination of more than one assays, and that the detection of SARS-CoV-2 in wastewater is genomic region dependent, as different results are being obtained by targeting different regions of SARS-CoV-2 genome.

### 2.4. Mutational Analysis of SARS-CoV-2 in Wastewater Using Targeted DNA-seq

**Figure 3:**
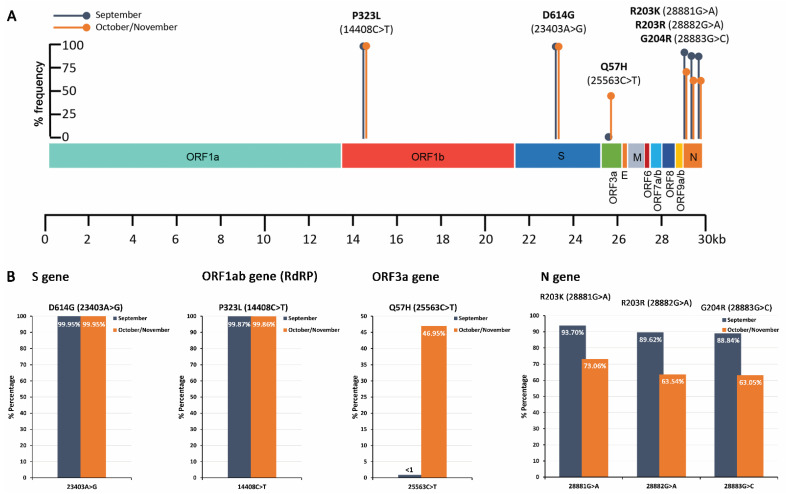
Schematic illustration on SARS-CoV-2 genomic map colored by genomic region (**A**) and bar plots (**B**) of the % frequency of D614G (S), P323L (RdRP), Q57H (ORF3a), R203K (N), and G204R (N) missense mutations in wastewater samples.

**Figure 4:**
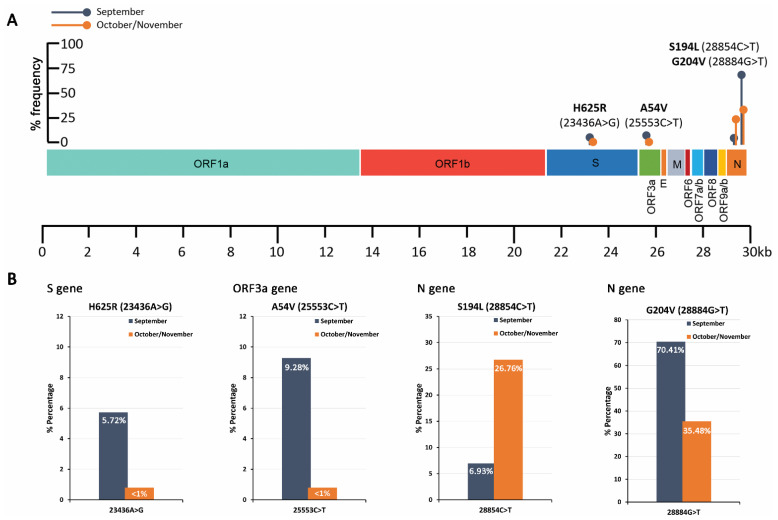
Schematic illustration on SARS-CoV-2 genomic map colored by genomic region (**A**) and bar plots (**B**) of the % frequency of H625R (S), A54V (ORF3a), S194L, and G204V (N) missense mutations in wastewater samples.

Five well-characterized missense mutations, D614G (23403A>G)-S gene, Q57H (25563G>T)-ORF3a gene, P323L (14408C>T)—ORF1ab/RdRP gene, R203K (28881G>A)—N gene, and G204R (28883G>C)—N gene, were initially targeted as proof-of-principle of our methodology to perform the analysis and quantification of SARS-CoV-2 mutations/strains in wastewater samples. To perform DNA-seq, novel nested PCR assays, against the above-mentioned point mutations, were designed and applied in September (n = 5) and October/November (n = 8) 2020 samples. The PCR products were used to generate DNA-seq barcoded libraries corresponding to September and October/November 2020 samples. The genomic variation profiling (list of existing SNVs or indels) of SARS-CoV-2 is summarized in Table 2, and visualized in Figure 3 and Figure 4.

The analysis highlighted that the G614 strain of SARS-CoV-2, originating from the D614G (23403A>G) point mutation in S gene, was exclusively detected (>99.9%) over the D614 strain. This finding is in line with the genomic data of the GISAID (https://www.gisaid.org/ accessed on 1 May 2021) and Nexstrain (https://nextstrain.org/sars-cov-2 accessed on 1 May 2021) databases, highlighting the significant prevalence of the G164 strain in percentage of >98% and ~100% worldwide and in Europe, respectively. Similarly, the P323L (14408C>T) substitution in the ORF1ab/RdPR gene was also prevalent, ~99.9%, in both time periods of sampling, also in agreement with the genomic epidemiology data (~99% worldwide and in Europe). Interestingly, the Q57H (25563G>T) mutation in the ORF3a gene was solely found observed in October/November samples at a percentage of ~47%, which is in accordance with the growing trend observed worldwide during the last months (11/2020, ~43%).

**Figure 5:**
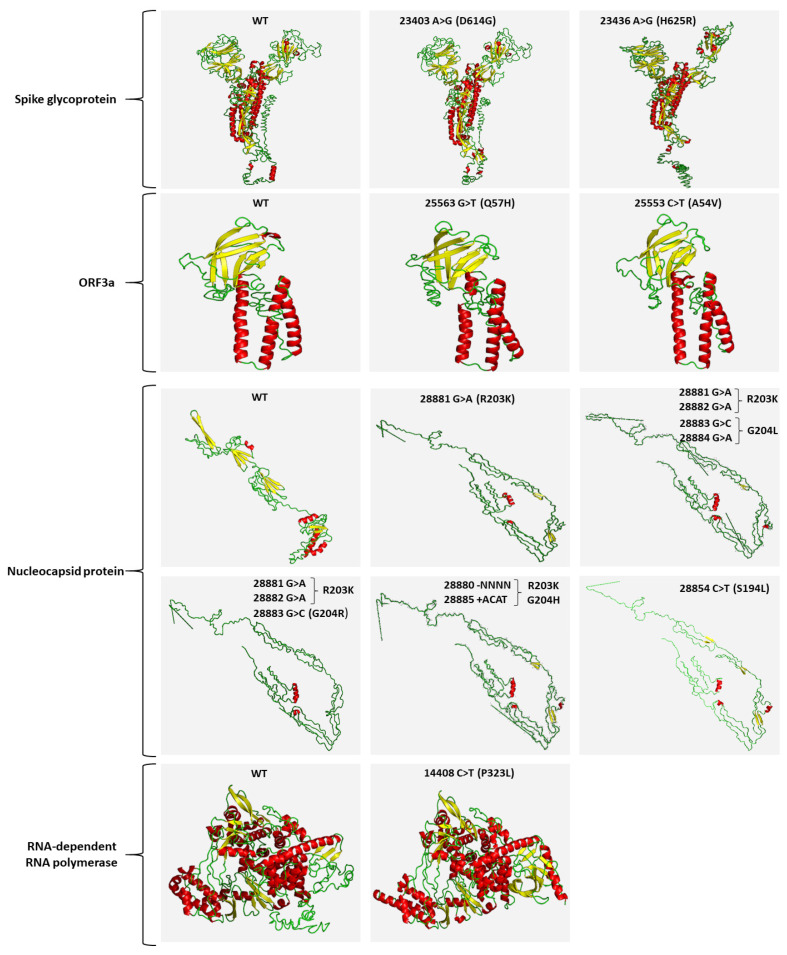
Predicted 3D structure models of “wild-type” and mutated SARS-CoV-2 proteins, using the I-TASSER v.5.1 server. For each protein, only the 3D structure with the highest confidence score is shown. Helix, sheet, and loop structures for each protein are depicted with red, yellow, and green colors, respectively.

In addition to the characterized D614G mutation, a previously unknown point mutation within S gene, H625R (23436A>G), was found at a frequency of 5.7% in September samples. The 23436A>G substitution results in the change of histidine to arginine at position 625 of the spike protein. Even though two similar amino acids are substituted, based on the in silico protein structure analysis, H625R leads to subtle alterations in the spike protein folding (Figure 5). As a result, the H625R-mutant spike protein may exhibit differential biochemical properties, which should be further investigated, since they may have a severe impact on the functionality of the protein, making the virus more transmissible and/or infectious. Structures of Spike trimeric are publicly available from the Protein Data Bank (PDB) database (https://www.rcsb.org/ accessed on 1 May 2021). Moreover, a novel point substitution, A54V (25553C>T), was also detected at a percentage rate of ~9% of September samples, resulting in the change of alanine to valine at position 54 of the ORF3a polypeptide. The amino acids both represent aliphatic, nonpolar neutral residues, and thus are not expected to induce critical structural alterations in ORF3a polypeptide functionality.

Focusing on the N gene, a declining trend was observed for the missense point mutations 28881G>A (R203K) and 28883G>C (G204R), as well as the synonymous substitution 28882G>A, from ~90% in September to ~70% in October/November samples. Our data agree with the declining trend of the above-mentioned substitutions worldwide (09/2020, 45% and 11/2020, 28%), although their absolute percentage in Greek samples remain significantly higher. Interestingly, a point substitution 28884G>T, which has been observed in ~1% worldwide, was overrepresented in our datasets, 09/2020, ~70% and 10–11/2020, ~35%. More importantly, our analysis highlighted the significant correlation of 28884G>T and 28883G>C point mutations, resulting in a novel amino acid substitution from glycine to leucine in position 204 (G204L) as compared with the single 28883G>C (G204R) or 28884G>T (G204V) substitutions. Moreover, the sequencing data revealed the existence of a simultaneous 4nt deletion following position 28880 (28881_28884del) and a 4 nt insertion at position 28885 (28885_28886insACAT). These two simultaneous indels lead to the R203K and G204H missense mutations of the nucleocapsid protein. The findings obviously indicate that R203K co-exists with G204R, G204L, or G204H variations. Although these mutations are located on the linker region (LKR) of the nucleocapsid phosphoprotein, which spans from position 175–254aa, only R203K belongs to the LKR’s crucial Ser/Arg (SR)-rich motif that contains putative phosphorylation sites [17]. Consequently, the absence of R203 residue is expected to have an immediate impact on the N protein folding and functionality, while any of the variations in position 204 of the protein (G204R, G204L, or G204H) lead only to subtle modifications (Figure 5).

Finally, a significant growing trend from 7% in September to 27% in October/November samples was revealed for the missense mutation S194L (28854C>T), in line with a similar trend observed worldwide (09/2020, 13% and 11/2020, 21%). Similar to R203K, the S194L mutation is also located on the SR-rich motif of the N protein and involves substitution of the hydroxylic neutral serine with the aliphatic neutral leucine. Since this region regulates the N protein oligomerization upon phosphorylation, the S194L could have a significant impact on protein function; this notion is in accordance with the dramatic changes in the predicted protein structure, and therefore merits further study.

**Figure 6:**
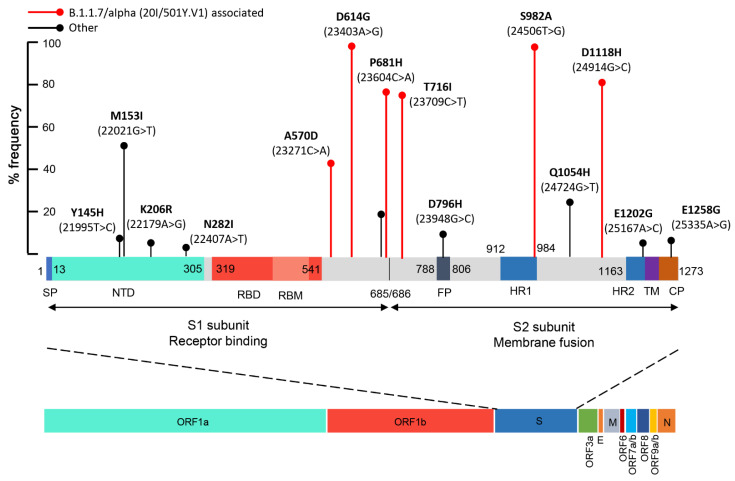
Schematic illustration of % frequency of missense mutations across the entire S gene colored by domain. Domains that are not visualized in the final map are colored grey. Numbers indicate amino acid residues. Β.1.1.7/alpha (20I/501Y.V1) associated missense mutations are indicated by red. SP, signal peptide; NTD, N-terminal domain; RBD, receptor-binding domain; RBM, receptor-binding motif; FP, fusion peptide; HR1, heptad repeat 1; HR2, heptad repeat 2; TM, trans transmembrane domain; CP, cytoplasmic peptide.

To further validate our methodology and to support the need for in-depth epidemiological analysis of existing and/or new emerging variants of SARS-CoV-2, we targeted the mutational analysis of the Spike (S) gene of SARS-CoV-2. In this regard, novel nested PCR assays, against the whole S gene (~4 Kb), were designed and applied in wastewater samples obtained daily in March 2021. The PCR products were used to generate a DNA-seq barcoded library corresponding to March 2021. The analysis led to the conduct of 3.13 million sequencing reads (Figure S3), with a median read length of 307 bp. The findings of these analyses are summarized in Table 3 and Figure 6.

According to the genetic markers (SNVs) observed (Table 3), only the Β.1.1.7/alpha (20I/501Y.V1) variant of concern (VOC) was detected, while the other VOCs, including B.1.351/beta (20H/501.V2), P.1/gamma (20J/501Y.V3), and B.1.617.2/delta (21A/S:478K), or variants of interest (VOI), were not detected in March 2021 samples from WWTP of Attica. According to the % frequencies of the genetic markers A570D (23271C>A), D614G (23403A>G), P681H (23604C>A), T716I (23709C>T), S982A (24506T>G), and D1118H (24914GG>C), the Β.1.1.7/alpha lineage VOC was detected in 80.6% ± 8.3 (mean ± SE), (median of 80.8%) of the total sequencing reads. Interestingly, the A570D (23271C>A) substitution was observed in a significantly lower percentage, i.e., 44.41%. According to the National Public Health Organization (NPHO) press releases and the data deposited to GISAID and analyzed by CoVariant (https://covariants.org/per-variant accessed on 20 July 2021) (Figure S4) regarding the analysis of clinical samples, the Β.1.1.7/alpha VOC frequencies in Greece in early March, end of March, end of April, and end of May 2021 were ~86%, 90%, 82%, and 73%, respectively. Although not safe to draw conclusions by the analysis of only a short period of time, our sequencing data on raw wastewater sample are in line with the prevalence of Β.1.1.7/alpha VOC at the same period in Greece and support the further validation of SARS-CoV-2 variant profiling in wastewater samples as a leading indicator of the variants’ spread at community/population levels.

## 3. Discussion

Considering the lack of effective treatments against COVID-19 [6], accurate, massive, and representative at the community level screening along with in-depth epidemiological analysis of existing or new emerging variants, are currently the only ways for an evidence-based approach of applying restriction measures in the near future. To this end, WBE of SARS-CoV-2 has been raised as a modern approach for real-time population-wide surveillance of SARS-CoV-2. Indeed, concentrations of SARS-CoV-2 in wastewater seemingly correlate with COVID-19 infection rates, and precede epidemic expansion and molecular testing at general population levels [14,18]. Moreover, SARS-CoV-2 genetic analysis in human samples could provide a severity-stratification tool for COVID-19 patients, as well as a much-needed approach for the early identification of newly emerging SARS-CoV-2 variants; nonetheless, these approaches remain particularly costly and time-consuming. Thus, mutational profiling of SARS-CoV-2 in wastewater could represent an innovative, cost-effective approach for the monitoring of existing variants and an early-warning system for new emerging ones, at community/population levels.

Τhe current analytical protocols for WBE of SARS-CoV-2 suffer from reduced sensitivity and false negative results, due to, for example, low viral loads, RNA degradation, and PCR inhibition [16]. To overcome these drawbacks, we performed RNA stability analysis of the SARS-CoV-2 RNA genome and identified highly predicted stable regions. This knowledge was exploited for the design of novel in-house methods that combining random hexamers-based reverse transcription and nested PCR/real-time PCR amplification against four highly stable regions of SARS-CoV-2 RNA. The evaluation of our novel assays highlighted the improved LOD (up to two copies/PCR reaction) as compared with one-step RT-PCR methods [19,20], and the significantly improved sensitivity as compared with in-house CDC/2019-nCoV_N1-based assay. Interestingly, more than half of the positive samples were detected by using only one assay, highlighting the anticipated on-going degradation of SARS-CoV-2 RNA in wastewater and clearly demonstrates that SARS-CoV-2 RNA detection in wastewater is genomic region dependent.

Previously, nested PCR approaches have been successfully applied for the detection of SARS-CoV-2 in wastewater [21,22,23,24]. We have documented, here, that the detection of SARS-CoV-2 in wastewater is assay dependent. In this regard, the reduction of false negative results in WBE requires the targeting of more than one SARS-CoV-2 genomic region. Based on our findings, the detection sensitivity of a single assay ranged from ~30% to 60%, while the combination of two or three assays improved sensitivity to 82% and 94%, respectively.

Mutation rates and genetic diversity of RNA viruses are significantly high, providing selective advantage to evolve and adapt to dynamic changes of environments and hosts [25]. Despite the presence of genetic proofreading machinery in SARS-CoVs [26], genetic diversity of SARS-CoV-2 is ever-growing, highlighting the unique position of CoVs in the RNA virus world. Thus, specific amino acid changes in SARS-CoV-2 encoded polypeptides could alter SARS-CoV-2 life cycle, infectivity, and/or antigenicity resulting in weakening the on-going vaccination programs and COVID-19 treatment efficacy.

As the SARS-CoV-2 Spike protein prevails as the main target of COVID-19 vaccines [27], mutations of S gene are frequently reported and studied [8,28]. In this regard, the D614G (23403A>G) missense mutation, which was initially identified in Europe, has emerged as the dominant pandemic form, likely due to a significant fitness advantage. The D614G mutation has been strongly associated with higher upper track viral loads and higher rates of younger hosts’ infection, as well as with increased replication and higher pseudotyped viral titers ex vivo [7,29,30]. Moreover, recent studies have confirmed the enhanced infectiveness and transmission of G614 strain in vivo [31,32]. Additionally, mutations of RNA-dependent RNA polymerase (RdRP), which is targeted by the anti-viral nucleoside analogues remdesivir [33] and favipiravir [34], are also in the spotlight. Interestingly, the P323L (14408C>T) mutation has been reported to co-evolve with D614G worldwide; this adaptation of the virus might strengthen SARS-CoV-2 G614 strain replication rates and infectivity [35].

In this regard, we have initially targeted five well-characterized missense mutations spanning different genomic regions of SARS-CoV-2, i.e., D614G (23403A>G), P323L (14408C>T), Q57H (25563G>T), R203K (28881G>A), and G204R (28883G>C), and specific nested PCR amplicons were sequenced using DNA-seq. This approach allowed the quantification of SARS-CoV-2 mutations/variations, and our data highlighted the significant prevalence (>99%) of D614G and P323L mutations in wastewater samples obtained from WWTP of Athens, Greece, during September-November 2020, in line with worldwide data based on COVID-19 patient samples. Additionally, the reported worldwide growing trend of the Q57H mutation was confirmed in our samples, with a percentage of ~47% in samples collected during October/November 2020.

Interestingly, a previously unknown missense mutation in the S gene, H625R (23436A>G), was identified in ~6% of September samples. Although H625R involves the substitution of two amino acids with positively charged polar side chains, the in silico structure analysis suggested significant changes in S protein folding. In this regard, specific monitoring of H625R along with other newly emerging mutants in COVID-19 patients and wastewater is necessary to conclude on their potential selective advantage and possible association with SARS-CoV-2 infectivity and effect on existing vaccines.

Focusing on N gene, the declining trend of 28881G>A, 28882G>A, and 28883G>C substitutions was confirmed in our samples. Interestingly, the 28884G>A substitution, which reportedly has a ~1% prevalence worldwide, was overrepresented in Athen’s samples (09/2020, 70% and 11/2020, 35%) and correlated significantly with 28883G>C, resulting in the G204L substitution of nucleocapsid protein. Moreover, a novel variant originating from a simultaneous 4 nt deletion (28881_28884del) and a 4 nt insertion at position 28885 (28885_28886insACAT), was observed in our samples, leading to the R203K and G204H missense mutations of nucleocapsid protein.

Finally, to facilitate the in-depth epidemiological analysis of existing and/or new emerging SARS-CoV-2 variants, we have expanded our methodology towards the mutational profiling of the whole S gene of SARS-CoV-2. The analysis of March 2021 samples as proof-of-principle of the methodology, highlighted that the Β.1.1.7/alpha variant was dominant in wastewater samples from WWTP of Attica, a finding in accordance with the prevalence of Β.1.1.7/alpha variant during the third pandemic wave in Greece.

## 4. Materials and Methods

### 4.1. SARS-CoV-2 RNA Stability Analysis

Τhe in silico stability analysis of SARS-CoV-2 RNA was carried out with the ScanFold algorithm [36]. The Wuhan-Hu-1 reference genome (NC_045512.2) was analyzed with ScanFold-Scan, using a 300 nt window with a 150 nt nucleotide step size, resulting in 198 analyzed windows. Each window was analyzed using the RNAfold algorithm that is included in the ViennaRNA package. For each window the minimum free energy (MFE) ΔG° structure and value was predicted using the Turner energy model at 18 °C.

### 4.2. Wastewater Sampling

The 24 h composite influent wastewater samples were collected from the WWTP of Athens (serves a population equivalent of 5,200,000 inhabitants). Influent wastewater samples were collected in precleaned high-density polyethylene (HDPE) bottles, transported on ice to the laboratory and stored at 4 °C. All the collected samples were analyzed immediately after the arrival at the laboratory.

### 4.3. Sample Concentration and RNA Extraction

Sample concentration was performed immediately after arrival using Polyethylene Glycol 8000 (PEG 8000, Promega Corporation, Madison, WI, USA) precipitation. In particular, 50 mL of an influent wastewater was centrifuged at 4750× *g* for 30 min at 4 °C to remove debris, bacteria, and large particles. The supernatant was transferred in a clean centrifuge tube, containing 3.5× g PEG and 0.8 g NaCl, mixed at ambient temperature until completely dissolved, and centrifuged at 10,050× *g* for 2 h, at 4 °C. Most of the supernatant was discarded without disturbing the pellet and the tube was centrifuged at 10,050× *g* for 5 min, at 4 °C. Finally, the pellet was reconstituted by 500 μL nuclease-free water.

RNA extraction was performed, by 200 μL concentrate using a Water DNA/RNA Magnetic Bead kit (IDEXX Laboratories Inc., Westbrook, Maine, USA) immediately following concentration. The % recovery was calculated by examining three concentrations, 10, 100, and 1000 copies/μL of the EURM-019 SARS-CoV-2 reference material, equal to 78.6%, 82.4%, and 91.3%, respectively [16,37]. Moreover, mengovirus (MgV) was used as an extraction control to evaluate extraction efficiency.

### 4.4. First-Strand cDNA Synthesis

Total RNA template from wastewater samples was reverse transcribed in a 20 μL reaction containing 5.0 μL RNA, 1.0 μL of 10 mM dNTPs mix (Jena Bioscience GmbH, Jena, Germany), 100 U SuperScript III reverse transcriptase (Invitrogen, Carlsbad, CA, USA), 50 U RNaseOUT recombinant ribonuclease inhibitor (Invitrogen), and 1.0 μL of 50 μΜ random hexamers (Invitrogen). The mixture of total RNA, dNTPs, and random hexamers was incubated at 65 °C for 5 min, while the reverse transcription took place at 25 °C for 10 min, followed by 50 °C for 50 min. Enzyme inactivation was performed at 70 °C for 15 min. The AMPLIRUN SARS-CoV-2 RNA control (Vircell S.L., Granada, Spain) was used as the SARS-CoV-2 complete genome control.

### 4.5. Detection of SARS-CoV-2

Novel nested PCR and nested real-time PCR assays were designed and validated against the identified highly stable regions of SARS-CoV-2 RNA. The Wuhan-Hu-1 reference genome (NC_045512.2) was used for the in silico analysis and design of SARS-CoV-2 specific primers and fluorescent probes (Table S1).

A Veriti 96-well fast thermal cycler (Applied Biosystems, Carlsbad, CA) was used for the nested PCR assays. The 25 μL of the reaction consisted of 5.0 μL cDNA template (1st PCR) or 2.0 μL PCR product (2nd PCR), 1.0 μL of 10 mM dNTPs mix (Jena Bioscience GmbH), 500 nM of each forward/reverse primer, and 1 U of Kapa Taq polymerase (Kapa Biosystems, Inc., Woburn, MA, USA). The thermal protocol consisted of polymerase activation step at 95 °C for 3 min, followed by 15 cycles (1st PCR) or 40 cycles (2nd PCR) of denaturation at 95 °C for 30 s, primer annealing at 60 °C for 30 s and extension at 72 °C for 1 min, followed by a final extension step at 72 °C for 5 min. After the completion of the 2nd reaction, 10 μL of PCR product was electrophoresed on 1.5% *w/v* agarose gel, visualized with ethidium bromide staining, and photographed under UV light.

The probe fluorescent-based real-time PCR assays were performed in a 7500 Fast Real-Time PCR System (Applied Biosystems). The PCR product of the 1st conventional PCR, as described above, were used as a template for the real-time PCR assay (2nd reaction). The 20 μL reaction consisted of 2.0 μL PCR product, 10 μL Kapa Probe Fast Universal (2X) qPCR Master Mix (Kapa Biosystems), 500 nM of each of the forward, reverse primers, and 125 nM of fluorescent probe. The thermal protocol included an initial polymerase activation step at 95 °C for 3 min, followed by 40 cycles of denaturation at 95 °C for 15 s, and finally the primer/probe annealing and extension step at 60 °C for 1 min.

### 4.6. Targeted DNA-seq for the Mutational Analysis of SARS-CoV-2 in Wastewater

In-house developed targeted DNA-seq assays, using semi-conductor sequencing technology were performed for the analysis of SARS-CoV-2 variations/mutations in wastewater samples.

Nested PCR assays were carried out to amplify the target regions. The Wuhan-Hu-1 reference genome (NC_045512.2) was used for the in silico analysis and the design of specific primers for the analysis of:

- The five missense mutations, D614G (23403A>G)-S gene, Q57H (25563G>T)-ORF3a gene, P323L (14408C>T)—ORF1ab/RdRP gene, R203K (28881G>A)—N gene, and G204R (28883G>C)—N gene (Table S2);
- The whole Spike (S) gene of SARS-CoV-2 (Table S3).

The Veriti 96-well fast thermal cycler (Applied Biosystems) was used for the nested PCR assays. The 25 μL of the reaction consisted of 5.0 μL cDNA template (1st PCR) or 2.0 μL PCR product (2nd PCR), 1.0 μL of 10 mM dNTPs mix (Jena Bioscience), 500 nM of each forward/reverse primer, and 1 U of Kapa Taq polymerase (Kapa Biosystems). The thermal protocol consisted of polymerase activation step at 95 °C for 3 min, followed by 20 cycles (1st PCR) or 40 cycles (2nd PCR) of denaturation at 95 °C for 30 s, primer annealing at 60 °C (most assays) or 57 °C (assays 5, 6, 10, and 14 of S gene analysis, Table S3) for 30 s and extension at 72 °C for 1 min, followed by a final extension step at 72 °C for 5 min. After the completion of the 2nd reaction, 10 μL of PCR product were electrophoresed on 1.5% *w/v* agarose gel, visualized with ethidium bromide staining, and photographed under UV light.

An Ion Xpress Plus Fragment Library Kit (Ion Torrent, Thermo Fisher Scientific Inc., Waltham, MA, USA) was employed for the construction of the DNA-seq library, using 1 μg of purified PCR product mix as input. Adapter ligation, nick-repair, and purification of the ligated DNA were carried out, based on the manufacturer’s guidelines. The adapter-ligated library was quantified using the Ion Library TaqMan Quantitation Kit (Ion Torrent) in an ABI 7500 Fast Real-Time PCR System (Applied Biosystems).

The sequencing template was generated with emulsion PCR on an Ion OneTouch 2 System (Ion Torrent), using the Ion PGM Hi-Q View OT2 kit (Ion Torrent). Next, the Ion OneTouch ES instrument (Ion Torrent) was used for the downstream template enrichment procedure. Ultimately, semi-conductor sequencing methodology was carried out in the Ion Torrent PGM system for the sequencing of the amplicons.

### 4.7. Bioinformatics Analysis

Alignment of the sequencing reads to the reference genome was carried out using the Burrows-Wheeler Aligner (BWA-MEM) [38]. The BAM files were analyzed by the Integrative Genomics Viewer (IGV) v.2.8.12 software for the visualization and assessment of the alignment results.

To efficiently call variants from the derived NGS datasets, we used the iVar algorithm [39], which is designed to detect virus genomic variations (SNVs or indels) from amplicon-based sequencing assays, using the default parameters; iVar was also used to identify the corresponding codons and translate variants into amino acids, based on the SARS-CoV-2GFF file.

### 4.8. In Silico 3D Protein Folding Analysis

To examine the predicted structural modification caused by each detected genomic variant, 3D structure models were generated with the I-TASSER v.5.1 server [40]. For the detection of the structural differentiations between the mutated and corresponding “wild-type” polypeptides, only the 3D structure with the highest confidence score was taken into consideration.

## 5. Conclusions

We report, here, that SARS-CoV-2 detection in wastewater is assay/genomic region dependent, and that significantly improved detection specificity was achieved by the combination of novel nested PCR/real-time PCR assays against highly stable SARS-CoV-2 genomic regions. Furthermore, we adopted our nested PCR approach for SARS-CoV-2 mutational profiling and variant quantification in wastewater by targeted DNA-seq. Our analysis indicated the high prevalence of the D614G and P323L missense mutations within S and RdRP genes, respectively; the growing trend of Q57H mutation in ORF3a; and the overrepresentation of R203K with G204R or G204L mutations in nucleocapsid phosphoprotein. Moreover, our approach was successfully applied for the mutational profiling of the SARS-CoV-2 S gene in wastewater samples from March 2021, highlighting the prevalence of the Β.1.1.7/alpha variant, in line with the fact that the Β.1.1.7/alpha variant was dominant in clinical samples during the same period of the third pandemic wave in Greece. Further mutational/variant profiling of wastewater samples for a long period of time could clearly shed light on the value of a wastewater-based mutational/variant analysis serving as a leading indicator of SARS-CoV-2 variants spread at community/population levels. We anticipate that our novel methods will further support the global efforts for monitoring SARS-CoV-2 spreading, as well as for detecting the emergence of new viral variants.

## 6. Patents

Provisional patent applications entitled “Detection and mutational analysis of an RNA virus in an environmental sample” were filed by the National and Kapodistrian University of Athens, and the authors M.A., P.G.A., N.V., N.S.T. and A.S. on 11 February 2021 to the Hellenic Industrial Property Organisation (case number 231-0004089064) and on 22 March 2021 to the Patent Cooperation Treaty (PCT application number PCT/EP2021/057316).

## Figures and Tables

**Table 1:** C_T_ values of our novel nested real-time PCR assays and the CDC/2019-nCoV_N1-based assay in the wastewater samples that there detected positive for SARS-CoV-2.

Samples	CDC N1-based	N	NSP3	Helicase	ORF3a
S1	36.2	27.0	-	-	-
S2	-	28.0	-	26.2	26.8
S3	34.3	27.2	26.3	-	-
S4	-	-	-	26.6	-
S7	-	28.0	-	-	-
S9	-	26.5	26.4	26.7	27.3
S15	35.2	28.9	-	-	-
S16	-	26.7	25.6	25.9	-
S19	-	-	-	-	28.0
S20	-	-	24.9	-	-
S21	-	27.1	25.7	-	28.0
S22	-	-	25.4	-	-
S23	-	-	30.9	25.1	28.5
S26	-	-	26.1	-	-
S28	-	29.0	26.1	26.3	-
S29	36.7	-	-	25.9	-
S30	35.9	28.2	-	-	-
Positive	5/30	10/30	9/30	7/30	5/30
% Sensitivity	29.4%	58.8%	52.9%	41.2%	29.4%
Assay Combinations % Sensitivity					
N and NSP3		82.4%			
N, NSP3 and helicase		94.1%			
“-”: Not Detected					

**Table 2:** Genomic variation profile (SNVs and indels) of SARS-CoV-2 by targeted DNA-seq.

September 2020								
Position	Ref. Base	Alt. Base	Alt. Freq. (%)	Total Depth	Ref. Codon	Ref. AA	Alt. Codon	Alt. AA
P323L (14408C>T)	C	T	99.87	240,183	CCT	P	CTT	L
D614G (23403A>G)	A	G	99.95	236,549	GAT	D	GGT	G
H625R (23436A>G)	A	G	5.72	249,444	CAT	H	CGT	R
A54V (25553C>T)	C	T	9.28	240,436	GCT	A	GTT	V
S194L (28854C>T)	C	T	6.93	307,007	TCA	S	TTA	L
28880	A	−NNNN	7.95	337,302	NA	NA	NA	NA
R203K (28881G>A)	G	A	93.70	264,387	AGG	R	AAG	K
R203 (28882G>A)	G	A	89.62	255,837	AGG	R	AGA	R
G204R (28883G>C)	G	C	88.84	269,981	GGA	G	CGA	R
G204V (28884G>T)	G	T	70.41	263,960	GGA	G	GTA	V
28885	A	+ACAT	3.09	340,323	NA	NA	NA	NA
**October/November 2020**								
**Position**	**Ref. Base**	**Alt. Base**	**Alt. Freq.** **(%)**	**Total Depth**	**Ref. Codon**	**Ref. AA**	**Alt. Codon**	**Alt. AA**
P323L (14408C>T)	C	T	99.86	249,568	CCT	P	CTT	L
D614G (23403A>G)	A	G	99.95	563,339	GAT	D	GGT	G
Q57H (25563G>T)	G	T	46.95	342,018	CAG	Q	CAT	H
S194L (28854C>T)	C	T	26.76	507,486	TCA	S	TTA	L
28881_28884del	A	−NNNN	5.08	597,906	NA	NA	NA	NA
R203K (28881G>A)	G	A	73.06	453,201	AGG	R	AAG	K
R203 (28882G>A)	G	A	63.54	463,095	AGG	R	AGA	R
G204R (28883G>C)	G	C	63.05	490,594	GGA	G	CGA	R
G204V (28884G>T)	G	T	35.48	429,127	GGA	G	GTA	V
28885_28886insACAT	A	+ACAT	3.50	599,861	NA	NA	NA	NA

**Table 3:** Genomic variation profile (SNVs) within the S gene of SARS-CoV-2 by targeted DNA-seq.

Position	Ref. Base	Alt. Base	Alt. Freq. (%)	Total Depth	Ref. Codon	Ref. AA	Alt. Codon	Alt. AA
Y144 (21994T>C)	T	C	6.75	116,465	TAT	Y	TAC	Y
Y145H (21995T>C)	T	C	8.75	100,235	TAC	Y	CAC	H
M153I (22021G>T)	G	T	52.41	114,991	ATG	M	ATT	I
K206R (22179A>G)	A	G	6.51	71,676	AAG	K	AGG	R
N282I (22407A>T)	A	T	4.12	153,621	AAT	N	ATT	I
A570D (23271C>A)	C	A	44.41	286,530	GCT	A	GAT	D
D614G (23403A>G)	A	G	99.95	135,792	GAT	D	GGT	G
C649 (23509T>C)	T	C	3.12	103,991	TGT	C	TGC	C
S659 (23539A>G)	A	G	17.97	263,236	TCA	S	TCG	S
Q675H (23587G>T)	G	T	20.12	154,225	CAG	Q	CAT	H
P681H (23604C>A)	C	A	78.56	150,675	CCT	P	CAT	H
T716I (23709C>T)	C	T	77.62	152,037	ACA	T	ATA	I
D796H (23948G>C)	G	C	11.16	147,600	GAT	D	CAT	H
I934 (24364T>C)	T	C	3.56	174,369	ATT	I	ATC	I
L959 (24437T>C)	T	C	5.09	156,278	TTA	L	CTA	L
S982A (24506T>G)	T	G	99.72	178,323	TCA	S	GCA	A
Q1054H (24724G>T)	G	T	25.88	175,445	CAG	Q	CAT	H
D1118H (24914G>C)	G	C	82.96	176,660	GAC	D	CAC	H
F1156 (25030T>C)	T	C	3.07	94,796	TTT	F	TTC	F
E1202G (25167A>G)	A	G	6.56	77,473	GAA	E	GGA	G
E1258G (25335A>G)	A	G	7.80	253,421	GAA	E	GGA	G

## Data Availability

The datasets generated during and/or analyzed during the current study are available from the corresponding author on reasonable request.

## Supplementary Materials

Supplementary Materials are available online at https://www.mdpi.com/article/10.3390/ijms22168498/s1.
