# Supplementary material for: Novel Nested-Seq Approach for SARS-CoV-2 Real-Time Epidemiology and In-Depth Mutational Profiling in Wastewater
Source: Int J Mol Sci. 2021 Aug 7;22(16):8498. doi: 10.3390/ijms22168498 (PMC8395163; doi:10.3390/ijms22168498)
Supplement: Supplementary file 1 [file ijms-22-08498-s001.zip › ijms-1287131-supplementary.pdf]

International Journal of
Molecular Sciences

MDPI

Article

Supplementary Materials: Novel Nested-Seq Approach for SARS-CoV-2 Real-time Epidemiology and In-depth Mutational Profiling in Wastewater

Margaritis Avgeris, Panagiotis G. Adamopoulos, Aikaterini Galani, Marieta Xagorari, Dimitrios Gourgiotis, Ioannis P. Trougakos, Nikolaos Voulgaris, Meletios-Athanasios Dimopoulos, Nikolaos S. Thomaidis and Andreas Scorilas

Table S1. Primers and qPCR probes for the *in house* nested PCR/real-time PCR assays

Assay	Oligos	Sequence (5′ – 3′)	Product length
**N assay**			
External set (1st reaction)	Forward primer	TGGACTTCCCTATGGTGCTAACA	310 bp
	Reverse primer	CAGCAAAGCAAGAGCAGCATC	
Internal set (2nd reaction)	Forward primer	GCTGCAATCGTGCTACAACTTC	125 bp
	Reverse primer	TTTCTTGAACTGTTGCGACTACG	
	qPCR probe (FAM-BHQ1)	AACATTGCCAAAAGGCTTCTACGCAGAA	
**Helicase assay**			
External set (1st reaction)	Forward primer	CAGATGAGTTTTCTAGCAATGTTGC	355 bp
	Reverse primer	TATATCTGCTGTCGTCTCAGGCA	
Internal set (2nd reaction)	Forward primer	AGCTCTCTACTACCCTTCTGCTCG	83 bp
	Reverse primer	TTAATGCCTTCTCACATAGTGCATC	
	qPCR probe (FAM-BHQ1)	TATACAGCTTGCTCTCATGCCGCTGTTGA	
**NSP3 assay**			
External set (1st reaction)	Forward primer	AGGGCTGGTGAAGCTGCTAAC	328 bp
	Reverse primer	CAGGTGGTGCTGACATCATAACA	
Internal set (2nd reaction)	Forward primer	TGAGTTACTTGTTTCAACATGCCA	91 bp
	Reverse primer	AGGGTTGTCTGCTGTTGTCCAC	
	qPCR probe (FAM-BHQ1)	TTCTTGCAAAAGAGTCTTGAACGTGGTGTGT	
**ORF3a assay**			
External set (1st reaction)	Forward primer	TGAACATGACTACCAGATTGGTGG	192 bp
	Reverse primer	ACATGTTCTTCAGGCTCATCAACA	
Internal set (2nd reaction)	Forward primer	GGGAATCTGGAGTAAAAGACTGTGT	103 bp
	Reverse primer	TGTTCAACACCAGTGTCTGTACTCA	
	qPCR probe (FAM-MGB)	TCACTTCAGACTATTACCAGCTG	

Table S2. Primers for the targeted DNA-seq analysis

Assay	Oligos	Sequence (5′ – 3′)	Product length
**D614G (23403A>G) assay - S gene**			
External set (1st reaction)	Forward primer	CCACAGACACTTGAGATTCTTGACA	268 bp
	Reverse primer	CACCAATGGGTATGTCACACTCA	
Internal set (2nd reaction)	Forward primer	TGTTCTTTTGGTGGTGTCAGTGTT	151 bp
	Reverse primer	AACCTGTAGAATAAACACGCCAAGT	
**Q57H (25563G>T) assay - ORF3a gene**			
External set (1st reaction)	Forward primer	CAAGGTGAAATCAAGGATGCTACTC	245 bp
	Reverse primer	GCAACGAGCAAAAGGTGTGAGT	
Internal set (2nd reaction)	Forward primer	CACTCCCTTTCGGATGGCTTA	122 bp
	Reverse primer	AACAAAGTGAACACCCTTGGAGA	
**P323L (14408C>T) assay – ORF1ab/RdRP gene**			
External set (1st reaction)	Forward primer	TGTTAACTGTTTGGATGACAGATGC	158 bp
	Reverse primer	GCTCTCTGAAGTGGTATCCAGTTGA	
Internal set (2nd reaction)	Forward primer	TGCATTCTGCATTGTGCAAACT	117 bp
	Reverse primer	AGTTGAAACTACAAATGGAACACCA	
**R203K (28881G>A) & G204R (28883G>C) assay – N gene**			
External set (1st reaction)	Forward primer	CAACATTGCCAAAAGGCTTCTAC	221 bp
	Reverse primer	GCCTTTACCAGACATTTTGCTCTC	
Internal set (2nd reaction)	Forward primer	CAGTCAAGCCTCTTCTCGTTCC	160 bp
	Reverse primer	GCTCTCAAGCTGGTTCAATCTGT	

Table S3. Primers for the mutational analysis of pike (S) gene of SARS-CoV-2

Assay	Oligos	Sequence (5′ – 3′)	Alignment on NC_045512.2	Product length
**Assay 1**				
External set (1st reaction)	Forward primer	GCTGTTATGTCTTTAAAAGAAGGTCAA	21430-21456	481 bp
	Reverse primer	GGACTGGGTCTTCGAATCTAAAGTA	21886-21910	
Internal set (2nd reaction)	Forward primer	CAGAGTTGTTATTTCTAGTGATGTTCTTG	21516-21544	362 bp
	Reverse primer	AATCCAGCCTCTTATTATGTTAGACTTC	21850-21877	
**Assay 2**				
External set (1st reaction)	Forward primer	GGACCAATGGTACTAAGAGGTTTGA	21777-21801	425 bp
	Reverse primer	CACTAAATTAATAGGCGTGTGCTTAGA	22175-22201	
Internal set (2nd reaction)	Forward primer	CTGTCCTACCATTTAATGATGGTGTT	21807-21832	352 bp
	Reverse primer	CCATCAATATTCTTAAACACAAATTCC	22132-22158	
**Assay 3**				
External set (1st reaction)	Forward primer	GCGAATAATTGCACTTTTGAATATG	22049-22073	428 bp
	Reverse primer	GATTTCAACGTACACTTTGTTTCTGAG	22450-22476	
Internal set (2nd reaction)	Forward primer	TCTTATGGACCTTGAAGGAAAACAG	22087-22111	351 bp
	Reverse primer	GCACAGTCTACAGCATCTGTAATGGT	22412-22437	
**Assay 4**				
External set (1st reaction)	Forward primer	TCAGGTTGGACAGCTGGTGC	22328-22347	427 bp
	Reverse primer	CTGCATAGACATTAGTAAAGCAGAGATC	22727-22754	
Internal set (2nd reaction)	Forward primer	GGGTTATCTTCAACCTAGGACTTTTC	22363-22388	351 bp
	Reverse primer	GGAGACACTCCATAACACTTAAAAGTG	22687-22713	
**Assay 5**				
External set (1st reaction)	Forward primer	AGATTTGCATCTGTTTATGCTTGG	22598-22621	434 bp
	Reverse primer	AAGTAACAATTAAAACCTTCAACACCA	23005-23031	
Internal set (2nd reaction)	Forward primer	AACTGTGTTGCTGATTATTCTGTCCT	22640-22665	347 bp
	Reverse primer	GCCTGATAGATTTCAGTTGAAATATCTC	22959-22986	
**Assay 6**				
External set (1st reaction)	Forward primer	CAATCTTGATTCTAAGGTTGGTGGT	22879-22903	397 bp
	Reverse primer	TGTCAAGAATCTCAAGTGTCTGTGG	23297-23321	

Internal set (2nd reaction)	Forward primer	TTGTTTAGGAAGTCTAATCTCAAACCTT	22925-22952	355 bp
	Reverse primer	TAGTGTCAGCAATGTCTCTGCCA	23257-23279	
**Assay 7**				
External set (1st reaction)	Forward primer	AATTTCAACTTCAATGGTTTAACAGG	23180-23205	429 bp
	Reverse primer	CCGAGGAGAATTAGTCTGAGTCTGA	23584-23608	
Internal set (2nd reaction)	Forward primer	ACTGAGTCTAACAAAAAGTTTCTGCC	23219-23244	347 bp
	Reverse primer	GCACCAATGGGTATGTCACACTC	23543-23565	
**Assay 8**				
External set (1st reaction)	Forward primer	TGGCGTGTTTATTCTACAGGTTCTA	23459-23483	438 bp
	Reverse primer	GGTGTTTTTGTCTTGTTCAACAGC	23873-23896	
Internal set (2nd reaction)	Forward primer	AATAGGGGCTGAACATGTCAACA	23512-23534	345 bp
	Reverse primer	CGGTTTAATTGTGTACAAAAACTGC	23832-23856	
**Assay 9**				
External set (1st reaction)	Forward primer	CCAGTGTCTATGACCAAGACATCAGTA	23744-23770	445 bp
	Reverse primer	CAGAAGTGTATTGAGCAATCATTTCA	24163-24188	
Internal set (2nd reaction)	Forward primer	ACTGAATGCAGCAATCTTTTGTTG	23801-23824	356 bp
	Reverse primer	AGCAAAGGTGGCAAAACAGTAAG	24130-24156	
**Assay 10**				
External set (1st reaction)	Forward primer	CTGGCTTCATCAAACAATATGGTG	24054-24077	427 bp
	Reverse primer	ATTGCACCAAAATTGGAGCTAAG	24458-24480	
Internal set (2nd reaction)	Forward primer	TTGCTGCTAGAGACCTCATTTGTG	24093-24116	343 bp
	Reverse primer	GCTTGTGCATTTTGGTTGACC	24415-24435	
**Assay 11**				
External set (1st reaction)	Forward primer	GATTGCCAACCAATTTAATAGTGCT	24328-24352	433 bp
	Reverse primer	AGTCACATGCAAGAAGACTACACCA	24735-24760	
Internal set (2nd reaction)	Forward primer	AAGACTCACTTTCTTCCACAGCAAG	24366-24390	350 bp
	Reverse primer	GGACATAAGATGATAGCCCTTTCC	24692-24715	
**Assay 12**				
External set (1st reaction)	Forward primer	TTAATTAGAGCTGCAGAAATCAGAGC	24596-24621	416 bp
	Reverse primer	TCCTTGAATGAGTCTAATTCAGGTTG	24986-25011	

Internal set (2nd reaction)	Forward primer	TGTCAGAGTGTGTACTTGGACAATCA	24648-24673	338 bp
	Reverse primer	CAAAGGATCATAAACTGTGTTGTTGA	24960-24985	
**Assay 13**				
External set (1st reaction)	Forward primer	CACAAAGGAATTTTTATGAACCACA	24876-24900	447 bp
	Reverse primer	AGCAGGATCCACAAGAACAACAG	25300-25322	
Internal set (2nd reaction)	Forward primer	ATTTGTGTCTGGTAACTGTGATGTTG	24922-24947	360 bp
	Reverse primer	CAACTGGTCATACAGCAAAGCATAA	25257-25281	
**Assay 14**				
External set (1st reaction)	Forward primer	TTGGCTAGGTTTTATAGCTGGCTT	25210-25233	380 bp
	Reverse primer	TTGAGGGTTATGATTTTGGAAGC	25567-25589	
Internal set (2nd reaction)	Forward primer	GCTTGATTGCCATAGTAATGGTGA	25230-25253	308 bp
	Reverse primer	CAACAATAAGCCATCCGAAAGG	25515-25537	

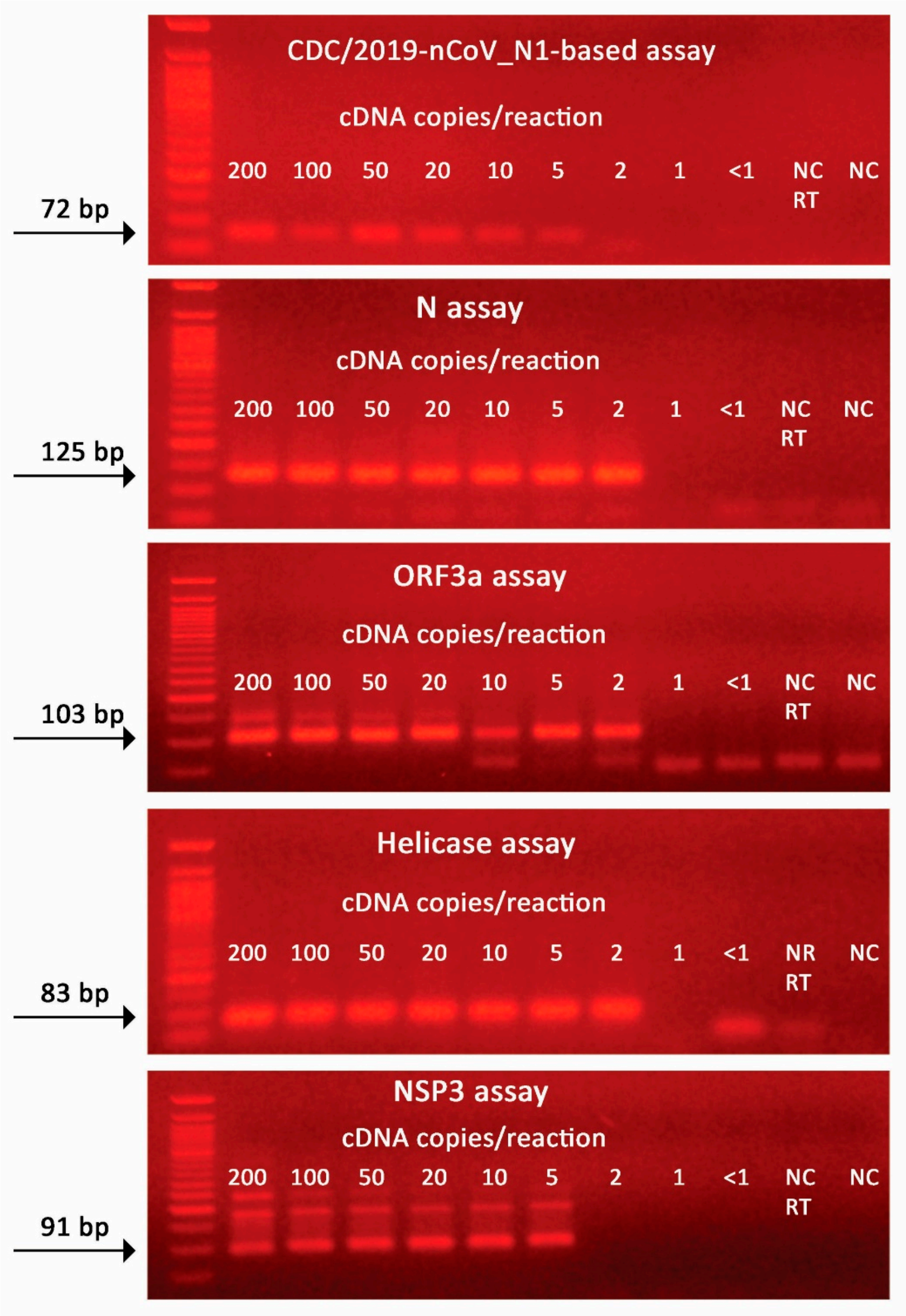

Figure S1. Agarose-gel electrophoresis of the PCR products of the in house CDC/2019-nCoV_N1-based assay and our nested PCR assays, A. N assay, B. ORF3a assay, C. Helicase assay and D. NSP3 assay, against serial dilutions of SARS-CoV-2 complete genome RNA control. M: 50 bp DNA ladder, PC: positive control, NC: PCR negative control, NCRT: reverse transcription negative control.

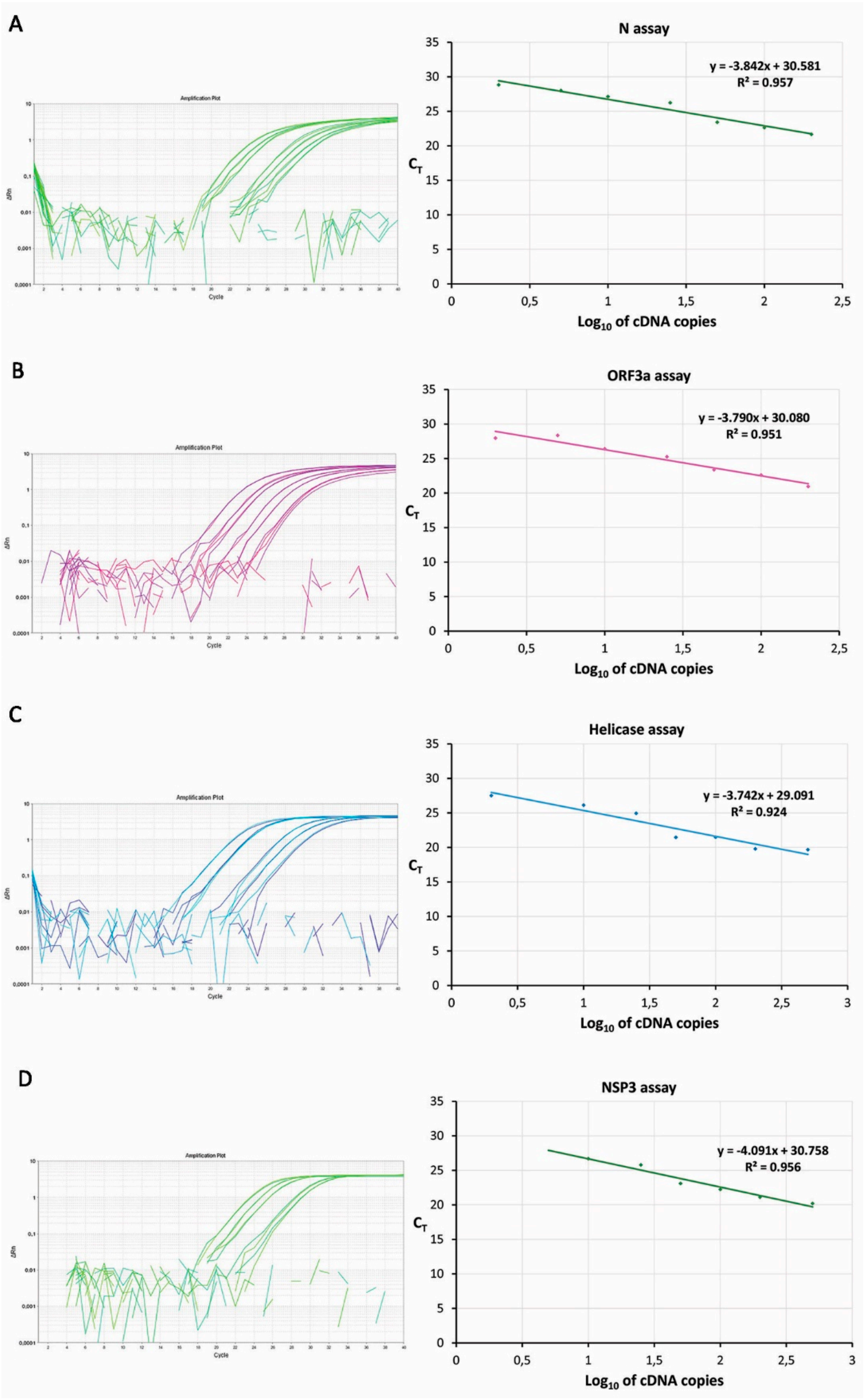

Figure S2. Amplification plots (left) and standard curves (right) of our developed nested real-time PCR assays: A. N assay, B. ORF3a assay, C. Helicase assay, D. NSP3 assay, against serial dilutions of SARS-CoV-2 complete genome RNA control.

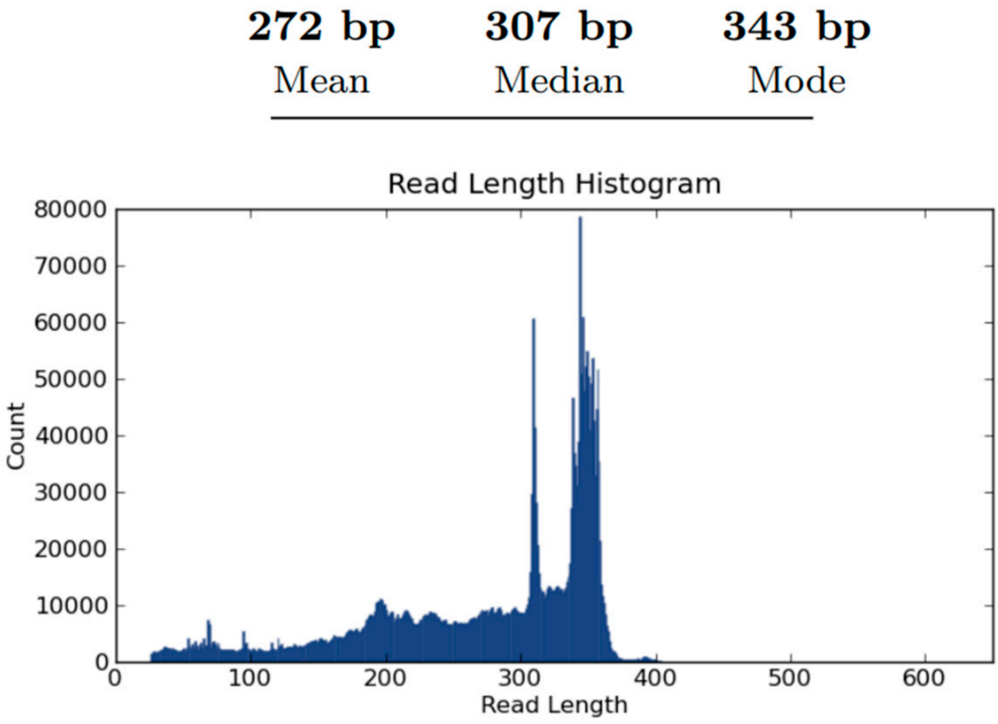

Figure S3. Histogram of sequencing reads length of S gene targeted DNA-seq in March 2021 samples.

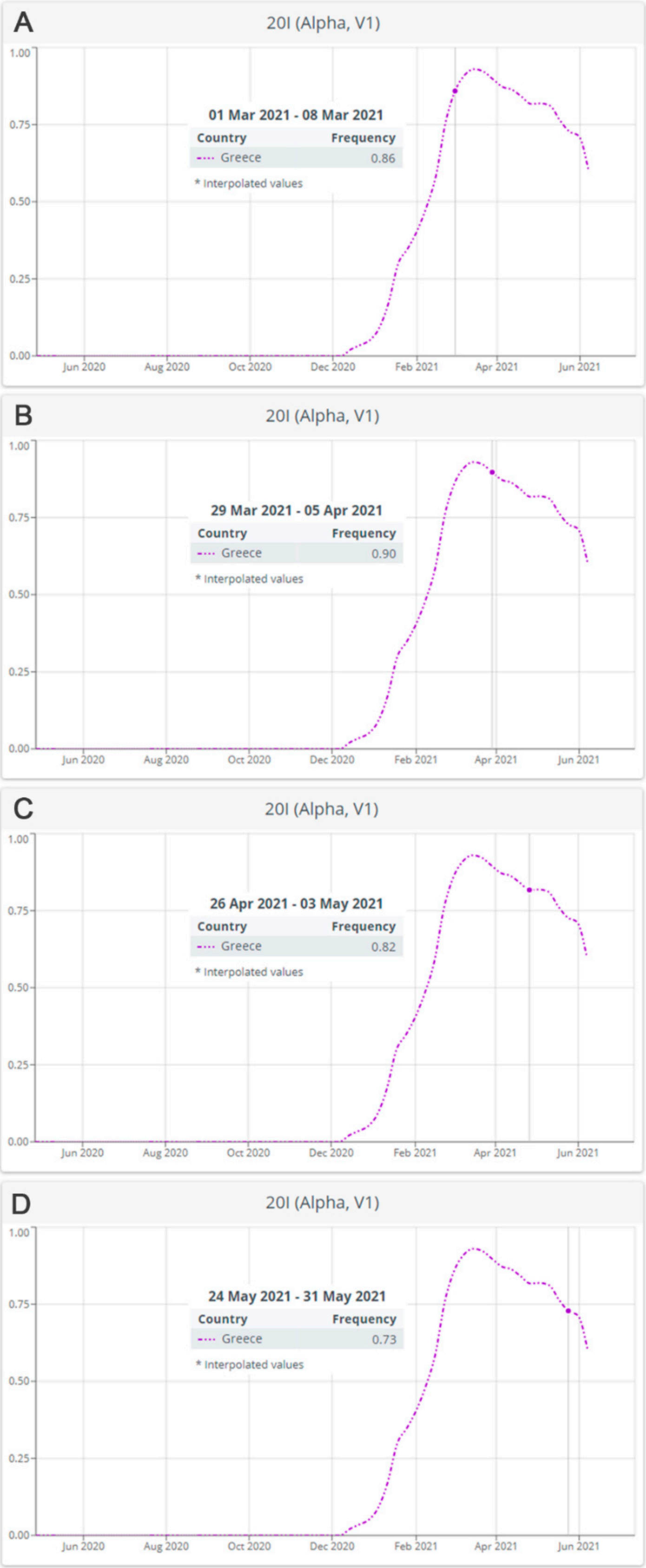

Figure S4. Frequencies of B.1.1.7/alpha (20I/501Y.V1) variant of concern in Greece clinical samples during early March (A), end of March (B), end of April (C) and end of May (D) 2021. Plots and analysis are provided by CoVariant (https://covariants.org/per-variant) using the data deposited in GISAID (https://www.gisaid.org/).

Publisher's Note: MDPI stays neutral with regard to jurisdictional claims in published maps and institutional affiliations.

Copyright: © 2021 by the authors. Submitted for possible open access publication under the terms and conditions of the Creative Commons Attribution (CC BY) license (https://creativecommons.org/licenses/by/4.0/).
